# Internal-Control Willingness and Managerial Overconfidence

**DOI:** 10.3389/fpsyg.2021.724575

**Published:** 2021-11-30

**Authors:** Bin Liu, Lin Li

**Affiliations:** ^1^School of Management, Hainan University, Haikou, China; ^2^School of Management, The University of Sheffield, Sheffield, United Kingdom

**Keywords:** willingness, managerial overconfidence, internal-control quality, internal-control willingness, internal control

## Abstract

Internal control is a branch of accounting subject, and accounting control and risk management are the core of enterprise internal control. Previous studies have shown that high-quality internal control inhibits or regulates managerial overconfidence (MOC). However, it is believed that the influential factors of internal-control quality (ICQ) are normally objective factors, such as corporate characteristics, financial status, and governance structure. Corresponding to another type of constituent element, that is, the subjective factor, which we called internal-control willingness, has not been explored. In this study, we defined *internal-control willingness* as the degrees of the subjective initiative of the internal-control construction and execution activities of enterprises. In addition, we proposed a method to measure internal-control willingness based on text analysis and principal component analysis using Python, and then, we tested its impact on ICQ and MOC. Our findings are as follows: (A) internal-control willingness has a positive impact on ICQ, and (B) internal-control willingness lowers MOC. Our study introduces subjective initiative factors into the field of internal control and also extends the understanding of internal-control theory. Based on empirical conclusions, we suggested that regulatory authorities and corporate boards improve incentive mechanisms to jointly strengthen the internal-control willingness of all employees, so as to help enterprise managers operate rationally.

## Introduction

Internal control is a branch of accounting subject, and accounting control and risk management are the core of enterprise internal control (COSO, 1992, 2004; Sarbanes and Oxley, 2002). At the same time, *Willingness*, as a term in psychology, is mainly used in the field of economics to measure the degree of the subjective will of actors, such as willingness to disclose financial information and willingness to pay (Hanemann, 1991; Elliott et al., 2014). *Internal-control willingness*, according to the psychological definition of *willingness* and its application in the accounting field, proposed in this study, is to measure the degrees of the subjective initiative of internal-control activities of enterprises. This subjective initiative, specifically, is the collective willingness of governance, management, and all employees (COSO, 1992; Ministry of Finance of China, 2008).

Does internal-control willingness matter? Ever since there has been any business firm, there has been internal control, which arises from the *willingness* of a business to engage in internal control so that it can succeed in the marketplace (Krishnan, 2005). As can be reasonably expected, this *internal-control willingness* varies from firm to firm. The importance of internal-control willingness and its variations are apparent even after many rules and regulations are codified (Ministry of Finance of China, 2008; COSO, 2013, 2017). For example, although the accounting rules and regulations governing all US firms are the same, Enron committed extremely serious mistakes (Deakin and Konzelmann, 2004). Thus, it must be that Enron had a different degree of internal-control willingness than other firms, which led to managerial overconfidence (MOC) and deliberately deviating from internal-control supervision.

Although internal-control willingness is important, it is difficult to observe. It is the reason why the studies in this field are almost blank. In traditional cognition, it is believed that the influential factors of internal-control quality (ICQ) are normally objective factors, such as corporate characteristics, financial status, and governance structure (Ashbaugh-Skaife et al., 2007; Doyle et al., 2007). The subjective initiative of internal control, that is, the internal-control willingness, has been ignored. Therefore, it is still necessary to explore whether the internal-control willingness of enterprises can affect MOC.

Our first contribution in this study is to introduce a methodology that quantifies internal-control willingness based on text analysis and principal component analysis (PCA) using Python. Empirical evidence shows that the internal-control willingness we designed has a positive impact on ICQ. Our second contribution is to show that, consistent with theoretical predictions, the internal-control willingness of enterprise lowers MOC. Our study introduces subjective initiative factors into the field of internal control and also extends the understanding of internal-control theory.

This study proceeds as follows. “Theoretical basis, literature review, and research hypotheses” section reviews previous literature, elaborates on the theoretical analysis, and develops research hypotheses. “Research design, variables, and data sources” section describes the research design, variables, and data sources. “Empirical results and analysis” section reports and analyzes empirical results. “Robustness tests” section reports various robustness checks. “Conclusion and discussion” section presents the conclusion and research implications.

## Theoretical Basis, Literature Review, and Research Hypotheses

### Theoretical Basis and Literature Review

Internal control is a branch of accounting subject, which is based on financial internal control. The earliest professional document involving “internal control” was the “Verification of Financial Statements” issued by the Federal Reserve Board (FRB) in 1929. In addition, the earliest definition of “internal control” was the “Examination of Financial Statements by Independent Public Accountants” issued in 1936. The U.S. COSO reports (COSO, 1992, 2004, 2013, 2017) are the pioneer of internal-control theory and are recognized by the Financial Accounting Standards Board (FASB) and the United States Securities and Exchange Commission (SEC).

In the field of practice, the “World Communications” accounting scandal in June 2002 completely dampened the confidence of investors in the capital market. To change this situation, the U.S. Congress and the government accelerated the passage of the Sarbanes-Oxley Act (SOX Act). An important innovation of the SOX Act is that companies listed in the U.S. stock market need to separately disclose the annual internal-control evaluation report and the internal-control audit report while disclosing the annual financial report and audit report. This bill highlights the important role of internal control in enterprise risk management. Since then, regulatory agencies around the world have drawn lessons from the U.S. COSO report and SOX Act to formulate internal-control systems and action plans applicable to each country, such as “Corporate Internal Control Basic Standards in China” (2008) and “Main Board Listed Companies Implement Corporate Internal-Control Standard System in Batches by Classification” in China (2012).

The U.S. COSO reports (COSO, 1992, 2004, 2013, 2017), the Sarbanes and Oxley (2002), and the Ministry of Finance of China (2008), all believe that *internal control* is implemented by the board of directors, board of supervisors, managers, and all employees of the company to achieve control goals. In addition, the goals of internal control are to “reasonably ensure the legality and compliance of business management, asset safety, financial reports, and related information that are true and complete, improve operational efficiency, and promote the realization of development strategies.”

Internal-control willingness is a variable tool, proposed in this study, to measure the degree of the subjective initiative of internal-control construction and execution. Therefore, research similar to this definition is almost blank. The closest research to this topic is the research experience on “*willingness.*” The “*willingness*” in applied psychology and behavioral economics is a variable of subjective initiative that can be measured, for example, “willing to pay,” and “willing to accept” (Coursey et al., 1987; Plott and Zeiler, 2005), “consumer willingness” (Lusk, 2003; Phelps et al., 2013), “Willing to participate” (Evans and Guthrie, 2006; Füller et al., 2010), etc. At the same time, the subjective initiative of internal-control construction activities is a verifiable and objectively existing natural phenomenon of applied psychology. It can be inferred that “internal-control willingness” is an accounting tool that can be identified, confirmed, and measured.

In academia, the measurement methods, economic consequences, and influencing factors of internal control are the focus of studies of scholars. Among them, the economic consequences of internal control, such as “firm risk,” “cost of equity,” “corporate governance,” “corporate cash holdings,” etc. (Ashbaugh-Skaife et al., 2009; Hoitash et al., 2009; Zheng and Chen, 2018), are of various types, but it is less relevant to our study. The measurement methods of internal control and the objective factors affecting ICQ are related to our study.

For one thing, the review of the measurement methods of internal control helps us to understand the optimal selection of ICQ. In previous studies, the results of internal control of a company are mainly reflected by the variable “ICQ” and measured according to the following methods: (1) internal-control effectiveness (Zhang et al., 2013; Liu and Wu, 2019). It is the final result of the annual internal-control evaluation report issued by the board of directors of the company. (2) Internal-control audit conclusion (Huang and Song, 2012; Liu et al., 2021). It is the verification conclusion given by the accounting firm on the corporate internal-control evaluation report. (3) Internal-control defects (Doyle et al., 2007; Ashbaugh-Skaife et al., 2009). It is an indirect content of internal-control information disclosure, and this type of information is not mandatory in China. (4) Internal-control index (ICI) (Lin B. et al., 2014; Chen et al., 2017). It is usually based on the specific content of the internal-control evaluation report, combined with the content of the annual financial report or other public information of the company. The ICI score is obtained by the self-designed evaluation system, and the advantage is that the score is a fine-grained measurement. In short, the first two methods belong to the direct evaluation of ICQ, and the data are derived from direct conclusions of the corporate official internal-control evaluation report or audit report. The latter two methods are indirect or derivative evaluations, and there is a lack of information on internal-control defects or doubts about the credibility of the index design.

For another, the review of objective factors affecting ICQ helps us to select control factors in model design. Since Doyle et al. (2007) explored the determinants of ICQ, scholars have focused on the objectively formed factors that affect ICQ, including first, corporate characteristics and financial status affect ICQ, which is manifested as enterprises with large scale, long creation times, and low financial risk have good objective resource advantages to achieve high ICQ (Ashbaugh-Skaife et al., 2007; Michelon et al., 2015). Second, the governance structure of the board of directors and audit committees affects ICQ, which is reflected in board size, ratio of independent directors, and performance of supervisors (Krishnan, 2005; Chalmers et al., 2019). Third, characteristics of corporate management, such as CEO and CFO, affect ICQ which is manifested in the factors such as professionalism, background, and rights (Hoitash et al., 2009; Lin Y. C. et al., 2014). In addition, some factors that come from outside of companies affect ICQ but have national differences or data limitations, such as characteristics of external auditors, national culture, regulatory, and market factors (Sarens and Christopher, 2010; Chen et al., 2016; Kanagaretnam et al., 2016).

In summary, in the field of economics, “*willingness*” is an instrumental variable that can be confirmed and measured. However, the study on “internal-control willingness” is in the ascendant. At the same time, the study of “internal-control willingness” on managerial confidence has not been carried out. The study contents above need to be demonstrated.

### Theoretical Analysis and Research Hypotheses

Before any formal regulation was mandated by any authority, businesses always had their own internal-control systems. Businesses are willing to voluntarily impose on themselves an internal-control system because it helps them succeed in the competitive marketplace (Krishnan, 2005). While this *internal-control willingness* has existed since the beginning of the existence of business, codified rules and regulations for internal control are only a recent phenomenon.

The U.S. COSO reports (COSO, 1992, 2004, 2013, 2017), the Sarbanes and Oxley (2002), and the Ministry of Finance of China (2008), all stated that the goals of the internal-control act include but are not limited to the following five aspects: “reasonably ensure the legality and compliance of business operation and management,” “ensure asset security,” “financial reports and related information are true and complete,” “improve operating efficiency and effectiveness,” and “promote the realization of development strategies for enterprises.”

The realization of the above goals depends on the dominant position of the board of directors in internal-control construction and also on the collective “willingness” of governance, management, and all employees. In addition, the collective “willingness” is based on the fact that internal control is a process implemented by the board of directors, board of supervisors, managers, and all employees to achieve internal-control goals (COSO, 1992; Ministry of Finance of China, 2008). The result of the realization of corporate internal-control goals is to be a success in the competitive marketplace and realize the development strategies. Therefore, there are the following logical chains among internal-control goals, subjective willingness, and final result of internal control (ICQ). Logically, internal-control willingness and ICQ have consistent goals. Thus, before proving the economic consequences of internal-control willingness, we first need to clarify the relationship between internal-control willingness and ICQ. We proposed a basic hypothesis as follows:

**Hypothesis 1:** Internal-control willingness has a positive impact on ICQ.

The notion of MOC (or optimism) is based on a stylized fact in social psychology known as the “better than average” effect (Weinstein, 1980; Weinstein and Klein, 1996; Ahmed and Duellman, 2013). Specifically, on the one hand, the first type of MOC is that managers (including directors, executives, and supervisors) systematically overestimate corporate returns or underestimate corporate risks, causing their behavior decisions to deviate from corporate returns (Heaton, 2002; Malmendier and Tate, 2005). On the other hand, the second type of MOC is that managers are in a position of salary advantage, which triggers their blind psychological self-confidence, causing their behavior decisions to deviate from corporate returns (Hayward and Hambrick, 1997; Brown and Sarma, 2007). It can be said that the measurement methods of MOC in existing studies can be included in the categories above.

The U.S. COSO reports (COSO, 1992, 2004, 2013, 2017), the Sarbanes and Oxley (2002), and the Ministry of Finance of China (2008), all have established five elements of “internal environment,” “risk assessment,” “control activities,” “information and communication,” and “internal supervision.” Among them, based on the first type of MOC, the internal-control system of enterprises relies on the “risk assessment” element to accurately identify internal and external risks, which is related to the realization of goals of the internal-control act. Meanwhile, the internal-control system of enterprises also analyzes and ranks the identified risks according to occurrence probability and comprehensive uses of risk response strategies, such as “risk aversion,” “risk reduction,” “risk sharing,” and “risk tolerance,” to achieve effective risk control. Then, all employees of enterprises actively construct a “risk assessment” system, help management face up to business risks, and prompt its behavioral decisions to conform to the goals of “asset security” and “improving operating efficiency and effectiveness” in the internal-control act. In other words, the active internal-control willingness of enterprises can help avoid the first type of MOC.

In addition, based on the second type of MOC, the internal-control system of enterprises relies on the “control activity” and “internal supervision” elements to restraint management decision-making authority and business behavior, through “authorization approval control,” “budget control,” “performance appraisal control,” “daily supervision,” and “special supervision.” Then, all employees of enterprises actively construct a “control activity” and “internal supervision” system, help management make reasonable decisions, compliant operations, and rational decisions, and promote their behavioral decisions to comply with the goals of “reasonably ensure the legality and compliance of business operation and management” and “promote the realization of development strategies for enterprises.” In other words, the active internal-control willingness of enterprises can help avoid the second type of MOC. Therefore, logically, internal-control willingness is negatively correlated with MOC. Therefore, this study proposes the second hypothesis as follows:

**Hypothesis 2:** Internal-control willingness lowers MOC.

## Research Design, Variables, and Data Sources

### Measurement of Internal-Control Willingness

*Internal-control willingness* is a tool, proposed in this study, to measure the degrees of the subjective initiative of internal-control activities of enterprises. To this end, we proposed the following technical route for measurement:

First, we found out voluntary pilot companies of internal control in all samples. In addition, those companies that voluntarily take the lead in the building internal-control system are most likely to have “positive” internal-control willingness. At the same time, its observation medium is the annual “Enterprise Internal-Control Self-Evaluation Report” (EICSER) approved and issued by corporate boards. Specifically, for our research, in the period of 2011–2016, we found out 188 enterprises that may have the “key features” (KFs) of “positive” internal-control willingness and a subtotal of 976 samples of annual EICSERs from the 6,386 overall research samples.

Second, we used the method of intelligent text analysis using Python to explore the KFs of positive internal-control willingness on the 976 samples of annual EICSERs above. We drew on the concept of Loughran and McDonald (2011) on English sentiment vocabulary classification, referred to the Chinese dictionary of “News Quantitative Public Opinion Database” in the CSMAR database (China Stock Market & Accounting Research Databases), and then identify the KFs in annual EICSERs that are related to “positive” internal-control willingness by means of manual labelings, such as “effective execution,” “increase value,” “sense of duty,” etc. Then, we aggregated a total of 45 groups of KFs and form a “bag of words” (BOW).

Third, we used the KF variables in the BOW, based on the “Traversal” function of Python, to obtain its statistical probabilities, and construct a word frequency matrix for text vectorization. Later, based on PCA using Python, we built an unsupervised PCA training algorithm model to carry out data dimensionality reduction on the contents of each EICSER. Specifically, we used the 45-dimensional data of the KFs in the BOW into 8-dimensional principal component data (*f1*,…, *f8*) through “dimensionality reduction” to achieve a cumulative contribution rate of 82.89% (>70%).

Fourth, we further calculated the “comprehensive value” (*Score*) of the text content of each EICSER and then, standardized the deviation of its value to map it to the [0, 1]. In the end, we achieved the measurement of the internal-control willingness value, which is marked as the variable *ICW*.

### Measurement of Managerial Overconfidence

This study draws on the following three methods to measure MOC from the following different perspectives:

The first method, based on the idea of Malmendier and Tate (2005), uses the relationship between managerial shareholding changes and year-end performance to measure MOC. Specifically, we used virtual variables: if managers accumulatively increase their shareholdings (total shareholding changes of directors, supervisors, and senior executives) and year-end net profit of a company is a loss or less than the value of the previous year, it is deemed that managers of the company are overconfident. In this case, the value is 1, and the others are 0. This variable is labeled as *MOC1*.

The second method, based on the idea of Hayward and Hambrick (1997), uses the relative ratio of managerial compensation to measure MOC. Hayward and Hambrick (1997) believed that the salary ratio of the highest managerial salary to the second, or the salary ratio of the top three managers to all managers, can measure MOC. Moreover, the greater the ratio, the greater the degree of MOC (Brown and Sarma, 2007). Specifically, for this study, we used the salary ratio of the top three managers to all managers, which is recorded as *MOC2*.

The third method, based on the method of Lin et al. (2005), uses the deviation degree between performance forecast and actual value to measure MOC. Lin et al. (2005) believed that the performance forecast of the financial report indicates managerial confidence. In addition, the difference between performance forecast and formal financial report can measure MOC. The greater the positive value of this difference, the greater the degree of MOC. Specifically for this study, we used the difference between the net profit of performance forecast and net profit disclosed in the actual financial report, and record it as *MOC3*.

### Main Test Models

The regression models of this study are based on the control factors confirmed by scholars in the field of influencing factors of internal control (Doyle et al., 2007; Hoitash et al., 2009) and influencing factors of MOC (Lang and Lundholm, 1996; Malmendier and Tate, 2005; Brown and Sarma, 2007; Ahmed and Duellman, 2013), refer to models (1) and (2) and Table 1 for details. *ICE, ICA*, and *ICI* represent one of the three measurement methods for *ICQ*. *MOC1*, *MOC2*, and *MOC3* represent one of the three measurement methods for *MOC*. *ICW* represents the internal-control willingness of enterprises.


(1) $$I\,C\,Q_{{}^{i\,t}}=\alpha_{0}+\alpha_{1}\,I\,C\,W_{{}^{i\,t}}+\alpha_{2}\,S\,i\,z\,e_{{}^{i\,t}}+\alpha_{3}\,R\,O\,I\,D_{{}^{i\,t}}+\alpha_{4}\,M\,S_{{}^{i\,t}}+\alpha_{5}\,L\,e\,v_{{}^{i\,t}}+\alpha_{6}\,B\,S_{{}^{i\,t}}+\alpha_{I\,n\,d}\,I\,n\,d_{{}^{i\,t}}+\alpha_{Y\,e\,a\,r}\,Y\,e\,a\,r_{{}^{i\,t}}+\varepsilon_{{}^{i\,t}}$$

(2) $$M\,O\,C_{{}^{i\,t}}=\beta_{0}+\beta_{1}\,I\,C\,W_{{}^{i\,t}}+\beta_{2}\,S\,i\,z\,e_{{}^{i\,t}}+\beta_{3}\,R\,O\,I\,D_{{}^{i\,t}}+\beta_{4}\,M\,S_{{}^{i\,t}}+\beta_{5}\,E\,d\,u_{{}^{i\,t}}+\beta_{6}\,P\,B_{{}^{i\,t}}+\beta_{I\,n\,d}\,I\,n\,d_{{}^{i\,t}}+\beta_{Y\,e\,a\,r}\,Y\,e\,a\,r_{{}^{i\,t}}+\varepsilon_{{}^{i\,t}}$$

**TABLE 1:** Variables of test models.

**Variable nature**	**Variable identification**	**Metric content**	**Variable description or calculation method**
Explained variable (*MOC*)	*MOC1*	Managerial overconfidence	Virtual variable: if managers accumulatively increase their shareholdings, and year-end net profit of a company is a loss or less than the value of the previous year, the value is 1, and others are 0.
	*MOC2*		Position of salary advantage, that is, the salary ratio of the top three managers to all managers.
	*MOC3*		Deviation degree of managers’ operating results, that is, the difference between net profit of performance forecast and net profit disclosed in actual financial report.
Explanatory variable	*ICW*	Internal-control willingness	Use text analysis and principal component analysis (PCA) by Python as measurement method, and the operation steps are detailed in section “Measurement of Internal-Control Willingness”
Comparison variables (*ICQ*)	*ICE*	Internal-control effectiveness	Virtual variable: internal control is effective as 1, and the others are as 0.
	*ICA*	Internal-control audit conclusion	Virtual variable: standard unqualified opinion is as 1, and the others are 0.
	*ICI*	Internal-control index	Refer to the “Internal-control Index” of the DIB database.
Control variables	*Size*	Corporate size	Natural logarithm of average total assets
	*ROID*	Ratio of independent director	The ratio of the number of independent directors to the total number of board members
	*MS*	Managerial shareholding	The sum of shareholding ratio of managers (directors, supervisors, and senior executives).
	*Lev*	Financial leverage	Financial leverage = total corporate debt/total assets
	*BS*	Board size	Natural logarithm of total number of directors
	*Edu*	Average educational level of managers	The mean value of managers’ academic qualifications. Among them, 1 for high school and below, 2 for junior college, 3 for undergraduate, 4 for master, and 5 for doctoral degree.
	*PB*	Average professional background of managers	The mean value of managers related to risk management. Among them, a manager is a risk management-related major, the value is 1, and others are 0.
	*Ind*	Industry	Primary industry classification
	*Year*	Year	Year value

### Research Samples and Data Sources

Our study uses China A-share main board listed companies as research samples. The Guidelines for Enterprise Internal Control, jointly issued by the China Ministry of Finance and the China Securities Regulatory Commission in 2010, stipulates that the Guidelines for Internal Control Evaluation of Enterprises and Guidelines for Internal Control Audit of Enterprises shall be implemented on the main board of the Shanghai Stock Exchange and Shenzhen Stock Exchange from January 1, 2012. Therefore, we selected the data of internal-control self-evaluation reports, annual financial reports, and audit report information from 2011 to 2016 as data sources.

At the same time, the samples are selected according to the following rules: (a) exclude financial and insurance companies since the financial statements of such listed companies have a special structure. (b) Exclude samples with data defects to ensure comparability. The data sources of this study include the Wind, CSMAR, DIB, Juchao Information, Shanghai, and Shenzhen Stock Exchange websites.

## Empirical Results and Analysis

### Descriptive Statistics

Table 2 reports the descriptive statistics. First, the mean value of the explanatory variable *ICW* is 0.38, which is between the median and the P75. It shows that from 2011 to 2016, only a small number of listed companies have strong internal-control subjective initiative. Second, among the explanatory variables, the mean value of the *MOC1* is 0.08, which shows that, in only 8% of the sample companies, managers accumulatively increase their shareholdings (the total shareholding changes of directors, supervisors, and senior management), and year-end net profit of the company is a loss or less than the value of the previous year. From this perspective, there are few companies whose managers are overconfident. Third, the mean value of *MOC2* is 0.44 and is close to the median value of 0.41, which shows that the total compensation of the top three managers accounts for 40% of the total compensation of all managers in A-share listed companies of China.

**TABLE 2:** Descriptive statistics results.

**Stats**	**N**	**Mean**	**SD**	**Min**	**P10**	**P25**	**Median**	**P75**	**P90**	**Max**
*MOC1*	6386	0.08	0.11	0.00	0.00	0.00	0.00	0.00	0.00	1.00
*MOC2*	6386	0.44	0.14	0.00	0.28	0.34	0.41	0.51	0.63	1.00
*MOC3*	3058^#^	–2.36	4250.51	–12664.10	–114.76	–22.28	–4.95	6.49	71.72	121563.00
*ICW*	6386	0.38	0.18	0.00	0.13	0.23	0.29	0.56	0.89	1.00
*ICE*	6386	0.80	0.40	0.00	0.00	1.00	1.00	1.00	1.00	1.00
*ICA*	6386	0.77	0.42	0.00	0.00	1.00	1.00	1.00	1.00	1.00
*ICI*	6386	638.13	163.59	0.00	541.95	618.04	672.08	711.60	750.93	995.36
*Size*	6386	22.60	1.45	17.76	20.88	21.66	22.44	23.42	24.51	28.51
*ROID*	6386	0.37	0.06	0.00	0.33	0.33	0.33	0.40	0.43	0.80
*MS*	6386	0.02	0.11	0.00	0.00	0.00	0.00	0.00	0.02	1.62
*Lev*	6386	52.01	20.13	0.71	24.20	37.34	52.94	67.74	77.84	103.73
*BS*	6386	2.18	0.21	0.00	1.95	2.08	2.20	2.20	2.40	2.89
*Edu*	6386	3.29	0.66	1.60	2.00	3.00	3.36	3.78	4.00	4.75
*PB*	6386	0.11	0.11	0.01	0.01	0.01	0.10	0.18	0.27	0.60

*^#^It should be noted that in the full sample of 6,386 companies, only 3,058 samples of companies disclosed performance forecasts before the official disclosure of financial reports. Therefore, the sample size of *MOC3* is 3,058.*

Fourth, the mean and median values of the *MOC3* are –2.36 and –4.95, respectively, which are relatively close to 0. At the same time, the value of the P10–P90 range is [–114.76, 71.72], which shows that the difference between the net profit of performance forecast and net profit disclosed in actual financial report is relatively close for a company, considering that the unit of measurement is RMB. However, the Min and Max values are –12664.10 and 121563.00, respectively, which shows that a small part of the sample has a large prediction bias, and managers are over self-confident or overconfident. Fifth, the mean values of the explanatory variables *ICE* and *ICA*, as same as comparison variables of the explained variable, are 0.80 and 0.77, respectively, indicating that internal-control levels of more than 3/4 listed companies are of high quality from 2011 to 2016. At the same time, the mean value of *ICI*s provided by the third-party DIB database is 638.13, which is between the P25 and the median. It shows that most listed companies have relatively high ICIs, only because some low-scoring companies, such as companies with a minimum value of “0,” lower the overall internal-control scores of listed companies.

### Basic Regression Analysis of Internal-Control Willingness on Internal-Control Quality

Panel A of Table 3 reports the Probit or OLS regression results among *ICW* on *ICE*, *ICW* on *ICA*, and *ICW* on *ICI*. The results show that *ICW* is both positively correlated with *ICE*, *ICA*, and *ICI* at the 1 or 5% significance level, indicating that internal-control willingness has a significant positive impact on ICQ. In detail, the stronger the internal-control willingness of a company, the higher the probability of “effective” in internal-control evaluation conclusion, the higher the rate to obtain “standard unqualified opinion” in internal-control audit conclusion, the higher the ICI score evaluated by the third-party organization DIB.

**TABLE 3:** Regression results.

**Panel A**	**(1)**	**(2)**	**(3)**	**Panel B**	**(4)**	**(5)**	**(6)**
**Variables**	***ICE***	***ICA***	***ICI***	**Variables**	***MOC1***	***MOC2***	***MOC3***
*ICW*	0.56***	0.33***	12.45**	*ICW*	–0.01***	–0.00**	–2.65*
	(4.12)	(3.15)	(2.36)		(–2.72)	(–2.41)	(–1.82)
*Size*	0.27***	0.25***	35.39***	*Size*	0.00***	0.03***	16.99***
	(10.12)	(10.32)	(21.72)		(2.81)	(19.77)	(3.05)
*ROID*	1.19**	0.41*	15.33**	*ROID*	–0.04**	–0.15***	–160.81*
	(2.07)	(1.79)	(2.32)		(–2.35)	(–4.64)	(–1.82)
*MS*	–0.84***	–0.25*	–65.81***	*MS*	0.01**	0.00***	90.89*
	(–3.01)	(–1.77)	(–2.89)		(2.32)	(3.21)	(1.73)
*Lev*	–0.00*	–0.01***	–1.65***	*Edu*	0.00***	0.00***	5.06*
	(–1.85)	(–3.78)	(–13.11)		(3.02)	(4.31)	(1.87)
*BS*	0.41***	1.04**	9.32*	*PB*	0.01**	0.00**	41.01
	(2.88)	(2.34)	(1.79)		(2.35)	(2.28)	(1.34)
*Constant*	–7.89***	–6.32***	–304.70***	*Constant*	0.02***	1.01***	287.32***
	(–10.41)	(–10.16)	(–6.47)		(5.10)	(23.54)	(3.21)
*Year & Ind*	Yes	Yes	Yes	*Year & Ind*	Yes	Yes	Yes
Observations	6,386	6,386	6,386	Observations	6,386	6,386	3,058
Pseudo R^2^/R^2^	0.29	0.21	0.12	R^2^	0.08	0.10	0.06
LR chi^2^/F	1783.67***	1521.33***	32.19***	F	19.03***	23.43***	12.99***

*p < 0.10; **p < 0.05; ***p < 0.01.

Among the control variables, most of the control factors related to ICQ are basically consistent with the conclusions of previous studies, including the following: (A) *size* is positively correlated with *ICE*, *ICA*, and *ICI* at the 1% significance level, similar to the conclusion of Doyle et al. (2007); (B) *ROID* and *BS* are positively correlated with *ICE*, *ICA*, and *ICI* at the 1–10% significance level, and Lev is negatively correlated with *ICE*, *ICA*, and *ICI* at the 10 or 1% significance level, similar to the conclusion of Hoitash et al. (2009); (C) MS is negatively correlated with *ICE*, *ICA*, and *ICI* at the 1 or 10% significance level, similar to the conclusion of Balsam et al. (2014).

The basic regression results confirm that internal-control willingness is an important factor to form the ICQ of a company, indicating that internal-control willingness, a new accounting measurement tool, can measure subjective initiative of internal control. Therefore, we believed that Hypothesis 1 can be accepted.

### Regression Analysis of Internal-Control Willingness on Managerial Overconfidence

Panel B of Table 3 reports the regression results between *ICW* and *MOC1* and *MOC2* and *MOC3*, which are three measurement methods of *MOC*. The results show that *ICW* is negatively correlated with *MOC1*, *MOC2*, and *MOC3* at 1, 5, or 10% significance level, indicating that internal-control willingness has a significant negative impact on MOC.

Among the control variables, most of the control factors related to MOC are basically consistent with the conclusions of previous studies, including the following: (A) *Size* is positively correlated with *MOC* at the 1% significance level, indicating that managers of large enterprises are more likely to be overconfident (Lang and Lundholm, 1996). (B) *ROID* is negatively correlated with *MOC* at the significance level of 1, 5, or 10%, indicating that the independent director mechanism can restrain MOC (Brown and Sarma, 2007). (C) *MS* is positively correlated with *MOC* at the significance level of 1, 5, or 10%, indicating that managers with a high shareholding ratio are more likely to produce overconfidence (Jensen, 1993). (D) *Edu* and *PB* are positively correlated with *MOC* at the significance level of 1, 5, 10, or close to 10%, indicating that highly educated managers or risk management professional managers are more likely to produce overconfidence. In addition, the background characteristics of managers above have a significant impact on their overconfidence (Hayward and Hambrick, 1997; Malmendier and Tate, 2005).

Therefore, we believed that Hypothesis 2 can be accepted, that is, internal-control willingness lowers MOC.

### Mechanism of Action and Enlightenment

In terms of the economic consequences of internal-control willingness, on the one hand, the mechanism by which internal-control willingness of enterprises has a negative impact on MOC is as follows:

Internal-control willingness is the willingness to construct and execute an internal-control system, which is one of the positive constituent factors of a high ICQ. According to the literature on positive organizational behavior (Luthans, 2002), “positive attitudes and mindsets of organizational actors → organizational tendency and behavior → realization of organizational (performance) goals” is a complete logical framework. Putting this logical framework in the field of internal control, the result shows “internal-control (positive) willingness → internal-control tendency and behavior → internal-control results (ICQ).” Thus, both logical and empirical evidence results indicate that internal-control willingness is an important component of internal-control results (ICQ).

At the same time, previous studies based on the data of Chinese listed companies have shown that high ICQ has an inhibitory or moderating effect on MOC (Xing and Song, 2015; Zheng and Chen, 2018). Therefore, as an important component of ICQ, internal-control willingness has a negative impact on MOC.

## Robustness Tests

### Balanced Panel Data

Due to the limitations in data sources, such as internal-control regulations and databases, this study adopts the pooled data method. To enhance the robustness and eliminate the impact of sample survival choices, this study converts the aforementioned pooled dataset into a balance panel set, which maintains the balance of individual samples of cross-section observations from 2011 to 2016. However, the dataset lost 2,696 observations through the conversion and yielded a new sample set with 3,690 observations. In particular, due to the data source limitation of the variable *MOC3* in Hypothesis 2, its sample size is further reduced to 1,686 observations.

Columns (1)–(3) of Table 4 report the regression results of the fixed effect (FE) model after the conversion from the pooled dataset to the balanced panel dataset. The results show that the correlation direction and significance of *ICW* on *MOC1*, *MOC2*, and *MOC3* are consistent with the main test. However, due to the loss of some observations, the significance of *ICW* in the three equations has decreased. Therefore, the results above show the advantage of using pooled data as the main test data.

**TABLE 4:** Robustness test results.

	**(1)**	**(2)**	**(3)**	**(4)**	**(5)**	**(6)**	**(7)**	**(8)**	**(9)**
**Variables**	***MOC1***	***MOC2***	***MOC3***	***MOC1***	***MOC2***	***MOC3***	***MOC1***	***MOC2***	***MOC3***
*ICW*	–0.01**	–0.00*	–2.63*	–0.01***	–0.00***	–2.66*	–0.01**	–0.00*	–2.23
	(–2.12)	(–1.89)	(–1.77)	(–2.89)	(–2.71)	(–1.90)	(–2.03)	(–1.88)	(–1.51)
*Size*	0.00***	0.03***	14.55*	0.00***	0.03***	17.80***	0.00***	0.03***	16.52***
	(2.98)	(7.76)	(1.77)	(2.98)	(20.15)	(3.12)	(2.79)	(18.98)	(2.99)
*ROID*	–0.04*	–0.10**	–141.32*	–0.05**	–0.16***	–169.54*	–0.04**	–0.14***	–157.73*
	(–1.72)	(–2.38)	(–1.78)	(–2.40)	(–4.87)	(–1.86)	(–2.31)	(–4.25)	(–1.83)
*MS*	0.01*	0.01**	85.51**	0.01**	0.00***	92.93*	0.01**	0.00***	89.12*
	(1.86)	(2.10)	(2.31)	(2.38)	(3.29)	(1.79)	(2.29)	(3.05)	(1.71)
*Edu*	0.00***	0.00***	4.67**	0.00***	0.00***	4.99*	0.00***	0.00***	5.01*
	(2.76)	(3.22)	(2.11)	(3.00)	(4.01)	(1.85)	(2.98)	(4.23)	(1.83)
*PB*	0.02**	0.00**	27.32	0.01**	0.00**	39.07	0.01**	0.00**	38.67
	(2.09)	(2.06)	(1.22)	(2.32)	(2.29)	(1.30)	(2.31)	(2.22)	(1.30)
*Constant*	0.03***	0.76***	220.19***	0.03***	1.12***	298.10***	0.02***	0.98***	280.12***
	(4.25)	(15.03)	(3.05)	(6.01)	(24.51)	(3.36)	(5.02)	(22.79)	(3.18)
*Year & Ind*	Yes	Yes	Yes	Yes	Yes	Yes	Yes	Yes	Yes
Observations	3,690	3,690	1,686	2,574	2,574	1,223	3,812	3,812	1,835
R^2^ (between)/R^2^	0.08	0.09	0.05	0.09	0.11	0.06	0.07	0.10	0.06
Wald Chi^2^/F	101.59***	132.01***	90.16***	19.98***	24.65***	13.08***	18.22***	23.02***	12.45***

*p < 0.10; **p < 0.05; ***p < 0.01.

### Sensitivity Test of Different Market Data Sources

The stock market in China is divided into two parts, namely, the Shenzhen Stock Exchange and the Shanghai Stock Exchange. The market positioning and service targets of the two exchanges are slightly different. To verify whether the empirical evidence obtained by the main tests is affected by different market data sources, which in turn affects the robustness of the test models and research conclusions, we performed grouping tests on the two markets. That is, we selected different samples from the Shanghai Stock Exchange and Shenzhen Stock Exchange and retested the verified Hypothesis 2.

Columns (4)–(6) of Table 4, respectively, report the OLS regression results of *ICW* on *MOC1*, *MOC2*, and *MOC3* in the Shenzhen Market group. The correlation direction and significance of *ICW* on *MOC1*, *MOC2*, and *MOC3* are basically consistent with the main tests. Columns (7)–(9) of Table 4, respectively, report the OLS regression results of *ICW* on *MOC1*, *MOC2*, and *MOC3* in the Shanghai Market group. Except for the results of *ICW* on *MOC3* in column (9), which are close to but not reaching the 10% significance level, the correlation direction and significance of the other two group results are similar to the main tests. Therefore, it can be considered that the empirical evidence obtained by the main tests is basically the same in different market groups.

In summary, the models and results are basically stable, and the conclusion of this study is still valid.

## Conclusion and Discussion

Internal-control willingness, proposed in this study, is to measure the degree of the subjective initiative of internal-control construction and execution. Meanwhile, we proposed a method to measure internal-control willingness based on text analysis and PCA using Python. Moreover, using the internal control and financial data from 2011 to 2016 in China, we also tested some effects of internal-control willingness on MOC. We obtained the following findings:

(A) Internal-control willingness can be identified, confirmed, and measured. Empirical evidence shows that the internal-control willingness we designed has a positive impact on ICQ. In detail, the stronger the internal-control willingness of a company, the higher the probability of “effective” in internal-control evaluation conclusion, the higher the rate to obtain “standard unqualified opinion” in internal-control audit conclusion, the higher the ICI score evaluated by the third-party organization DIB. Therefore, internal-control willingness, a new accounting measurement tool, can measure subjective initiative of internal control.
(B) Internal-control willingness lowers MOC. The mechanism is that internal-control willingness is one of the positive constituent factors of a high ICQ. Meanwhile, previous studies based on the data of Chinese listed companies have shown that high ICQ has an inhibitory or moderating effect on MOC (Xing and Song, 2015; Zheng and Chen, 2018). Therefore, as an important component of ICQ, internal-control willingness has a negative impact on MOC.

Based on empirical conclusions, our study puts forward the following research implications: subjective initiative factors play an important role in the construction and execution of corporate internal control. Therefore, it is recommended that regulatory authorities should issue favorable policies, and corporate boards should improve incentive mechanisms, to jointly guide and strengthen the internal-control willingness of all corporate employees. The overall increase of corporate internal-control willingness will help restrain the overconfidence behavior of individual managers, help the management face up to the business risks, and help the management make reasonable, compliant, and rational decision-making.

At the same time, our research also has some limitations: first, for the measurement of corporate internal-control willingness, we adopted a method based on text analysis and PCA. Although this method pioneered the measurement of “internal-control willingness,” other measurement methods still need to be explored. Second, the economic consequences of “internal-control willingness” may be numerous, and we only discussed its impact on MOC. Other economic consequences need to be discovered in follow-up studies.

## Data Availability Statement

The raw data supporting the conclusions of this article will be made available by the authors, without undue reservation.

## Author Contributions

BL: conception and design of the study, drafting the manuscript, and funding acquisition. LL: acquisition of the data and analysis and interpretation of the data. Both authors contributed to the article and approved the submitted version.

## Conflict of Interest

The authors declare that the research was conducted in the absence of any commercial or financial relationships that could be construed as a potential conflict of interest.

## Publisher’s Note

All claims expressed in this article are solely those of the authors and do not necessarily represent those of their affiliated organizations, or those of the publisher, the editors and the reviewers. Any product that may be evaluated in this article, or claim that may be made by its manufacturer, is not guaranteed or endorsed by the publisher.
